# Absorption and amplification singularities in metasurface etalons with gain

**DOI:** 10.1515/nanoph-2025-0085

**Published:** 2025-05-22

**Authors:** Nelson de Gaay Fortman, Georg-Michael Krause, Peter Schall, A. Femius Koenderink

**Affiliations:** Institute of Physics, University of Amsterdam, 1098 XH, Amsterdam, The Netherlands; Department of Physics of Information in Matter and Center for Nanophotonics, NWO-I Institute AMOLF, Science Park 104, 1098 XG, Amsterdam, The Netherlands

**Keywords:** metasurface, optical gain, singular optical response

## Abstract

Passive reflective metasurfaces can possess perfect absorption conditions: Singular scattering anomalies at which all impinging light is absorbed. Perfect absorption is a common yet powerful metasurface design option with applications in energy harvesting, sensing, and more. Less common is the inclusion of optical gain to the system, which can give rise to a singular condition for perfect amplification. We analyze absorption and amplification singularities in plasmon antenna metasurface etalons with gain with a simple transfer matrix model. Our etalon follows the Salisbury screen design: A metal ground plate spaced by dielectric medium from an array of resonant plasmonic scatterers. We include frequency dispersive models for gain media and discuss the limitations of time reversal symmetry arguments for relating gain singularity conditions (reflectivity poles) to the well-known perfect absorption conditions (reflectivity zeros) of metasurface etalons. We show that for metasurface etalons with both gain and loss, gain can induce both perfect absorption and gain singularities, and we describe topological constraints on their creation and annihilation. Our findings have implications for the fields of non-Hermitian photonics, parity-time symmetric scattering systems, and dynamically controllable active metasurface pixels.

## 1 Introduction

Reflective metasurfaces are of considerable interest for their ability to control the reflection amplitude, phase, and absorption of optical waves [1], [2], [3], [4], [5], [6], [7], [8], [9], [10], [11]. A common geometry is inspired by the radio frequency concept of Salisbury and Dallenbach screens: Thin (patterned) layers at a carefully chosen distance from a metal ground plate that achieve perfect absorption of impinging waves [10], [12], [13]. In optics, this motif of metasurfaces at quarter wavelength (or similar) distances from a mirror has led to advances in reflective metasurface pixels [2], [5], [7], [9], and has been used to turn intrinsically weakly absorbing layers like two-dimensional (2D) materials into effective photodetectors [14], [15], [16]. The seminal paper of Chong et al. relates perfect absorption to complex frequency plane analysis of the scattering matrix of photonic structures in terms of zeros and poles: The scattering matrix eigenvectors with eigenvalues 0 and *∞* [17]. In fact, according to Krasnok et al. [18] the scattering response of a system is completely determined by such zeros and poles. By adding losses one can bring zeros from the upper half complex frequency plane onto the real frequency axis. Conversely, poles correspond to scattering resonances, and when brought to the real frequency axis by gain engineering, they become amplification singularities [19], [20], [21]. This understanding explains Salisbury and Dallenbach screens, more general cases of coherent perfect absorption (CPA) [18], [22], [23], [24], [25], [26], [27], [28], [29], [30], as well as of CPA lasing [18], [19], [20], [30], [31], [32], [33], [34]. Zeros and poles furthermore have a topological character that expresses in the phase response [28]. Recently, active tuning of metasurface response through the control of zeros and poles has received interest in numerical studies. One focus has been on the active control over absorption singularities using thermo- or electro-optical mechanisms [35], [36], while another has been on amplification singularity tuning by means of optical gain [37], [38]. However, actively tuned singular response of a plasmon metasurface Salisbury screen with optical gain has received little attention.

**Figure 1:**
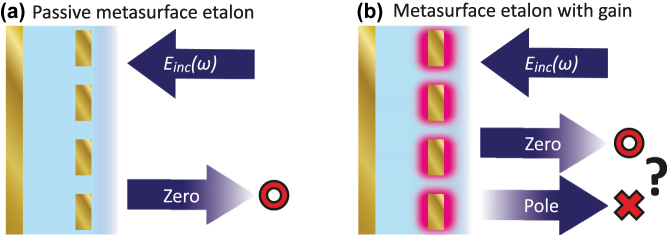
Singular reflection in metasurface etalons with loss and gain. (a) For a lossy, plasmon metasurface in front of a mirror, hybridization between the plasmon resonance and etalon resonances leads to pairs of perfect absorption conditions: Zeros in reflection accompanied by phase singularities in a parameter space spanned by frequency and etalon spacing. (b) When optical gain is included both reflection zeros (◦) and reflection poles ( × ) can arise.

In this work, we theoretically study perfect absorption (zeros) and amplification singularities (poles) in amplifying plasmon antenna metasurface etalons, extending the Salisbury-screen analogon for perfect absorption in such structures [10] to gain. The philosophy of the work is highlighted in Figure 1: While a passive metasurface etalon (panel a) may host pairs of reflection zeros we will show that the introduction of gain can give rise both to zeros and poles, and we analyze topological constraints on these singularities. We develop a transfer matrix model [39] for metasurface etalons with loss and gain and address the emergence of zeros and poles in dependence of (1) whether gain/loss is included in the spacer layer or in the metasurface, and (2) in the lattice case, how the gain is included in the meta-atoms. Furthermore, we emphasize the importance of the numerical models used for loss and gain. An appealing viewpoint comes from the field of time-reversal and Parity-Time (PT) symmetry, where nonresonant imaginary refractive index (*n*″) of equal magnitude but opposite sign are associated with time-reversal, *i.e.*, an interchange of loss and gain properties. This viewpoint implies simple relations between perfect absorption and gain singularity conditions. However, we argue that the physics is crucially affected by the need to account for the physical frequency dispersion in gain media: A correct gain dispersion means that gain and loss *are not* time-reversed equivalents through sign-inversion of the imaginary part of the dielectric response. Finally, we argue that Salisbury screens with gain and loss can show real-frequency zeros and poles that are very close in parameter space, which may be interesting for dynamically controllable amplitude and phase metasurface pixels with very large dynamic range.

## 2 Model

In this work, we consider semi-analytical modeling of amplifying metasurface etalons in which plasmon particle lattices are held in front of a mirror, and in which gain is introduced, either in the dielectric spacer or in the particle lattice. We start by reviewing simple physical models for loss and gain materials and scatterers, before recapitulating the transfer matrix method to calculate the stack response.

### 2.1 Model for loss materials and scatterers

The canonical model for a plasmonic particle with loss [40] starts with the Drude model for the free electron plasma, yielding the complex-valued dielectric constant

(1) $$\epsilon_{\text{Drude}}\left(\omega \right)=\epsilon_{\infty }-\frac{\omega_{p}^{2}}{\omega^{2}+i\omega \gamma_{p}},$$

where *ω*_*p*_ is the plasma frequency, and *γ*_*p*_ the Ohmic damping rate. When substituted into the Rayleigh expression for the quasi-static electric dipole polarizability of a subwavelength sphere of dielectric constant *ϵ* and radius *r* in a host of dielectric constant *ϵ*_host_ (we use the convention **p** = 4*πϵ*_host_ *ϵ*_0_ *α* **E** so that polarizabilities have units of volume), one finds

(2) $$\alpha_{0}\left(\omega \right)=r^{3}\frac{\epsilon \left(\omega \right)-\epsilon_{\text{host}}}{\epsilon \left(\omega \right)+2\epsilon_{\text{host}}}$$

displaying the well-known localized surface plasmon resonance at *ϵ*(*ω*) = −2*ϵ*_host_. When *ϵ*_*∞*_ = *ϵ*_host_, this resonance is exactly Lorentzian.

(3) $$\alpha_{0}=\frac{V\omega_{0}^{2}}{\omega_{0}^{2}-\omega^{2}-i\omega \gamma }.$$

**Figure 2:**
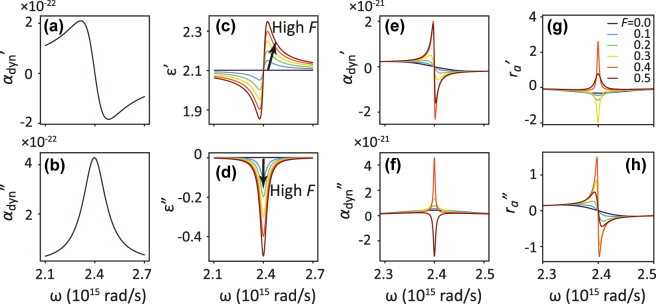
Response functions of lossy and gainy constituents of the plasmon metasurface etalons with gain. (a,b) Plot of Eq. (4): Real and imaginary part of the dynamic polarizability of a single plasmon rod of *V* = 3 × 10^−23^ m^3^ (Lorentz model fitted to COMSOL simulation from Ref. [28]). (c,d) Plot of Eq. (5): Real and imaginary permittivity of a model dielectric medium with a Lorentzian gain resonance frequency *ω*_*g*_ = *ω*_*p*_ = 2.4 × 10^15^ rad/s, line width *γ*_*p*_ = 0.01*ω*_*g*_ and population inversion parameter *F* = (0, 0.1, 0.2, 0.3, 0.4, 0.5). Note how compared to the plasmon dispersion not only the imaginary but also the real part is flipped in sign. (e,f) Plot of Eq. (4) with Eq. (5). Dynamic polarizability of a plasmon antenna coupled to a resonant gain bath according to the model of Manjavacas [50], effectively forming a combination of resonances (a,b) and (c,d). For increasing gain the sign of the metal dispersion remains, but from *F* = 0.5, Im(*α*_dyn_) flips sign. (g,h) Plot of Eq. (7): Reflectivity *r*_*a*_ of a subdiffractive (*a* = 350 nm) gain plasmonic metasurface in glass for increasing pump strength. Re[*r*_*a*_] flips sign as the singularity condition between *F* = 0.3 and *F* = 0.4 is traversed.

The particle resonance frequency $\omega_{0}=\omega_{p}/\sqrt{3\epsilon_{\text{host}}}$ and damping rate follow straightforwardly from the Drude parameters, while the oscillator strength is quantified by *V* (units of volume, equal to *r* ^3^ for the Rayleigh sphere). For strong scatterers, scattering into the far-field comes with an additional loss channel. It is well known in literature [41], [42] that a self-consistent theory for multiple scattering requires to include a radiation damping factor *i*2/3*k* ^3^

(4) $$\alpha_{\text{dyn}}\left(\omega \right)=\frac{1}{1/\alpha_{0}-i2/3k^{3}},$$

where *k* = *nω*/*c* is the wave number of the light in the medium surrounding the scatterer $\left(n=\sqrt{\epsilon_{\text{host}}}\right)$ [41], [42]. The resulting ‘dynamic’ polarizability satisfies the optical theorem, meaning that scattering equals extinction at zero Ohmic damping, while at non-zero Ohmic damping the extinction exceeds scattering, with the deficit equal to absorption. In this work, we start with parameters from [28], which make Eqs. (2) and (4) accurately fit finite element simulations of extinction and scattering of single nanorod plasmon antennas in glass (*n* = 1.45) for polarization along the long axis of the rods. These parameters in terms of Eq. (3) read *ω*_0_ = 2.4 × 10^15^ rad/s, damping rate *γ*_*p*_ = 9.3 × 10^13^ s^−1^ and *V* = 6.9 × 10^−23^ m^3^ for Au nanorods (100 × 50 × 40 nm^3^) in glass. We will vary *V* to control oscillator strength. Figure 2a and b show the resulting dynamic polarizability of the lossy plasmon particles. The real part shows the typical dispersive line shape, indicating the typical *π* phase slip in scattering that occurs upon crossing the resonance. The imaginary part is a positive Lorentzian line shape, and through the relation *σ*_ext_ = 4*πk*Im*α*_dyn_ directly indicates the resonance in the extinction cross section.

### 2.2 Extension to gain materials

To describe amplifying instead of lossy scatterers, it is tempting to simply reverse the sign of the damping rate, or equivalently complex conjugate *ϵ*. While such a transformation has been argued to be equivalent to time reversal [43], [44], [45], [46], [47], [48], we note that special care is required because scattering by a gainy particle is not the time-reverse of scattering by a lossy particle. Scattering redistributes light from a single input port (a plane wave) over all outgoing ports (outgoing spherical wave) at a rate given by the radiation damping term *i*2/3*k* ^3^. Time-reversing material loss into gain does not also redefine the input and output ports in a scattering problem, meaning that the radiation damping term does not change sign. Given these subtleties, it is useful to inspect gain susceptibility models and review the ramifications for scattering.

A common model in the laser community [49] to describe an inverted population of atoms in a dielectric medium is to add the susceptibility *χ*_gain_ of an ensemble of atoms in inversion to the background permittivity *ϵ*_*b*_(*ω*) as

(5) $$\epsilon \left(\omega \right)=\epsilon_{b}\left(\omega \right)+\chi_{\text{gain}}\left(\omega \right)$$

with a resonant Lorentzian lineshape

(6) $$\chi_{\text{gain}}\left(\omega \right)=F\frac{\gamma_{g}}{\omega -\omega_{g}+i\gamma_{g}},$$

where *ω*_*g*_ is the resonant frequency of the gain medium, *γ*_*g*_ its line width, and *F* quantifies the density of excited atoms (population inversion controlled by pump strength in experiments). This model has been proposed for 3- or 4-level gain atoms [49], [50], [51], [52], [53], [54]. Figure 2c and d plots Lorentzian susceptibility model *χ*_gain_ for atoms in glass, with *ω*_*g*_ = *ω*_*p*_ = 2.4 × 10^15^ rad/s and *γ*_*p*_ = 0.01*ω*_*g*_ for various pump strengths *F*. Importantly, the gain not only makes the imaginary part of the permittivity/polarizability negative, but also affects the dispersion in the real part of the response function. We note that a Drude scatterer can be transformed into an amplifying resonant scatterer with valid gain dispersion (as Figure 2c and d) not by *γ* → −*γ*, as an intuitive time-reversal argument would suggest, but instead by *V* → −*V*. Ref. [53] explains this behavior on basis of a quantum mechanical microscopic model of a 3-level atom upon pumping, from which the authors derive the classical dynamic polarizability of an atomic scatterer with gain. Below inversion, the atom dynamic polarizability displays a lossy Lorentzian polarizability, much like a plasmonic particle (panel a,b). Upon reaching population inversion, the atom becomes transparent. Crossing through transparency, the polarizability goes through zero, flipping sign both in its imaginary and real part. The sign flip in the imaginary part indicates negative extinction cross sections, i.e., amplification. The scattering cross sections *σ*_scatt_ = 8*π*/3*k* ^4^|*α*_dyn_|^2^ instead remain positive.

Several works have proposed including gain into lossy plasmonic scatterers [47], [48], [50], [54], [55], [56], [57], [58], [59], [60]. We follow the description of Manjavacas [50], which implements gain by taking the dielectric constant of a nanoparticle as *ϵ*(*ω*) = *ϵ*_Drude_(*ω*) + *χ*_gain_(*ω*), and evaluating Eq. (2) and Eq. (4). Figure 2e and f plot the real and imaginary part of the polarizability at various gain levels *F*. For low gain values, the polarizability is similar to that of the lossy plasmonic particle, sharpening with increasing gain as the intrinsic Drude loss is compensated. The dispersion then goes through a condition of strong scattering and strong positive extinction to a regime of negative extinction (net gain). For such a compound particle, the dispersion in the real part of polarizability *does not* flip sign.

Finally, we review how to convert the single particle polarizability into metasurface reflectivity. To calculate *r*_*a*_ for periodic arrays of identical scatterers, one can use Ewald lattice summation techniques that include retarded multiple scattering interactions in the point dipole approximation [42], [61]. For lattices consisting of identical scatterers specified by quasi-static polarizabilities *α*_0_, arranged in unit cells of area *A*, the reflectivity in the non-diffractive regime reads

(7) $$r_{a}\left(\omega \right)=\frac{2\pi ik}{A}\frac{1}{1/\alpha_{0}\left(\omega \right)-\frac{2\pi ik}{A}}.$$

Eq. (7) contains only the imaginary contribution of the lattice interaction term, as the real part induces a shift in resonance frequency which we incorporate into *ω*_0_ [28]. Interestingly, Eq. (7) replaces the single nanoparticle radiation damping correction that is appropriate for the single scatterer (Eq. (4)) with a collective lattice damping term 2*πik*/*A*. This lattice damping term increases with antenna density and signifies superradiant damping for coherently radiating dipole arrays. [28]. In the limit of high areal density, the reflectivity approaches a perfect mirror *r*_*a*_(*ω*) = −1. In Figure 2g and h, we examine complex reflectivity of a plasmonic metasurface with gain included in *α*_0_, according to the approach of Manjavacas [50]. The real value of *r*_*a*_ (Figure 2g) becomes increasingly negative with more gain, until it reaches the singular condition *α*_0_ = *A*/2*πik* (between *F* = 0.3 and 0.4), at which point the gain compensates the Ohmic and lattice radiation loss. At this point the reflectivity flips sign to large positive amplitudes, before converging to zero with further increase of *F*. Panel (h) shows that near the singularity condition, the Lorentzian curve of Im(*r*_*a*_) has the steepest slopes.

### 2.3 Metasurface etalon transfer matrix model

To describe the complete response of the composite system from the single material response just described, we use a transfer matrix method laid out in Ref. [39]. We consider normally incident radiation only. The transfer matrix method as introduced in the seminal work [62] relates parallel electric and magnetic (*E*, *H*) fields at the front side of a stack of a dielectric layer (*z* = 0) with those at the back side (*z* = *d*_stack_) via multiplication of characteristic matrices of individual layers: *M*_stack_ = *M*_*N*_ × *M*_*N*−1_…*M*_2_ × *M*_1_ (with *m* = 1, 2, …*N* enumerating the layers from front to back). From the stack matrix *M*_stack_, the complex reflection and transmission amplitudes follow as

(8) $$\left(\begin{array}{c} t\\ ikt \end{array}\right)=M_{\text{stack}}\left(\begin{array}{c} 1+r\\ ik\left(1-r\right) \end{array}\right).$$

Our stack will be composed of transfer matrices *M*_*d*_ for homogeneous layers of index *n* and thickness *d*, and a transfer matrix for the metasurface *M*_meta_. *M*_*d*_ is well known in literature, but *M*_meta_ should depend on *r*_*a*_ and merits careful attention. For non-diffractive metasurfaces, *M*_meta_ is obtained by assuming a zero thickness layer with reflection coefficient *r*_*a*_, and also assuming that both slabs immediately neighboring the metasurface have identical refractive index [39]. Explicitly, *M*_*d*_ and *M*_meta_ [39] read

(9) $$\begin{array}{ll} M_{d} & =\left(\begin{array}{cc} \cos kd & \frac{1}{k}\sin kd\\ -k\sin kd & \cos kd \end{array}\right)\;\text{and}\\ M_{\text{meta}}\left(r_{a}\right) & =\left(\begin{array}{cc} 1 & 0\\ \frac{2ikr_{a}}{1+r_{a}} & 1 \end{array}\right). \end{array}$$

The model does not allow for any diffraction channels, so we only consider lattices with subdiffractive pitches. We retrieve *r*_*a*_ from Eq. (7).

While we use the transfer matrix method for all calculations in this work, we note that for a simple two layer etalon (one mirror, one metasurface) one can extract an analytical Fabry–Perot formula [27], [28], [63]

(10) $$r_{\text{FPI}}=\frac{r_{a}+r_{m}\left(1+2r_{a}\right)e^{2{ik}_{0}nd}}{1-r_{a}r_{m}e^{2{ik}_{0}nd}}.$$

Here, *r*_*m*_ denotes the back reflector reflection coefficient. In this work, we use glass-backed gold mirrors with *n* = 0.25 + 3.46*i* and thickness 50 nm or 20 nm, and dielectric spacers of varying thickness and index *n* = 1.45. Solving Eq. (8) with proper *M*_*d*_, these values yield reflection coefficients *r*_*m*_ = −0.5935 − 0.6502*i* and −0.3066 − 0.5126*i*, respectively. Eq. (10) has been used in literature to explain the topological constraints on perfect absorption conditions in absorbing metasurface etalons [28].

## 3 Results

### 3.1 Gain equivalent of lossy metasurface

**Figure 3:**
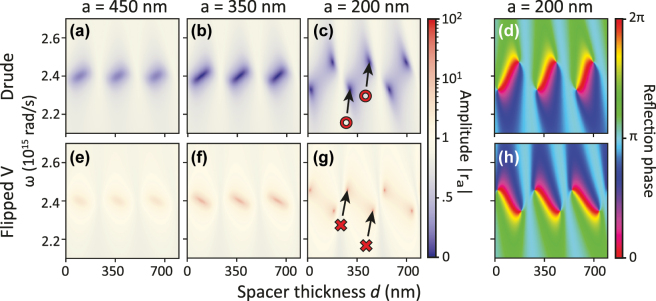
Transfer matrix calculations in (*ω*, *d*) space of an etalon with a regular Drude metasurface versus its amplifying equivalent. (a–c) Reflectivity amplitude for metasurfaces of increasing particle density (decreasing square arrays pitch *a* listed as plot titles), assuming standard lossy plasmon particles. At *a* = 200 nm, absorption zeros around the plasmon resonance appear, two of which are pointed out with a red ◦ for clarity. In reflection phase ((d), referenced to same structure without particles), the pairs of zeros appear as phase singularities of opposite topological charge ± 1. (e–g) Response for the amplifying equivalent structure (*V* → −*V* in Eq. (2)). Amplification singularity pairs occur at *a* = 200 nm (indicated with×). However, note that the response does not simply exchange loss for gain as also the dispersion around *ω*_0_ is inverted. Evaluated for *V* = 3 × 10^−23^ m^3^ and Au mirror thickness 50 nm.

We now discuss the response of Salisbury screens that include optical gain. We examine increasingly complex scenarios for metasurface etalons with gain, focusing on singular responses. The simplest case is presented in Figure 3: Reflectivity of a standard etalon where a lossy mirror is separated by a transparent spacer from a layer composed of nanoparticles whose response are defined by the Lorentzian Drude model (Eq. (3)). We compare the standard case of loss, modeled by positive oscillator strength *V* shown in panels (a-d), with the simplest self-consistent model of amplification of equivalent magnitude set by negative volume, *V* → −*V* in panels (e-h). Parameters are chosen to closely resemble the standard plasmonic Salisbury screen in Ref. [28], sketched in Figure 1a. The left three columns of Figure 3 show reflectivity amplitude in a phase space (*ω*, *d*) spanned by etalon spacing *d* and frequency *ω*, with different panels corresponding to successively higher antenna density (plots labeled with lattice pitch $a=\sqrt{A}$ , assuming square lattices). The rightmost column displays reflectivity phase. Throughout this work, we use color scales for |*r*_*a*_| that are linear from 0 to 1 (blue to white), and logarithmic for |*r*_*a*_| > 1 (white to red, clipped at *r*_*a*_ = 10^2^). Panels (a-d) show the case of a standard lossy metasurface Salisbury screen. The response is dominated by the hybridization of the antenna resonance at 2.4 × 10^15^ rad/s with etalon resonance conditions, especially visible in (c): At thicknesses of approximately 250, 500 and 750 nm, hyperbolic white features curve from top slightly rightward towards bottom. In between these etalon conditions, the reflectivity amplitude drops with increasing antenna density, signifying absorption. At *a* = 200 nm, the value of *r*_*a*_ has become large enough that points of perfect absorption occur. They arise in pairs, and coincide with singularities in the reflection phase shown in panel (d) (phase referenced to the phase pickup in absence of the particles). In the parameter space spanned by *ω* − *d*, phase singularities arise in pairs of opposite charge ± 1, indicating a 2*π* phase increment over a (counter)clockwise loop around the singular points. These charges highlight the topological nature of the singularity conditions.

Next, we turn to the amplifying counterpart (*V* → −*V*) in Figure 3e–h, the geometry of which is sketched in Figure 1b. At first glance, it appears that as loss is replaced by gain, the absorption features in reflection are replaced by enhanced reflection. At low antenna density the reflectivity enhancement is modest (mimicking the finite absorption in panels (a,b)), while above a threshold antenna density, the gain metasurface etalon displays poles in reflectivity. These emerge as the counterparts of perfect absorption in (c), and likewise correspond to phase singularities (compare panel h and d). While qualitatively the results for gain are clearly analogous to the case of loss (points of singular behavior, arising in pairs, exchanging zeros for poles), there is one striking difference: For pairs of perfect absorption the red-shifted (blue-shifted) singularity appears for spacing larger (resp. smaller) than the etalon condition, but in the case of gain this ordering is reversed. Associated with that, the phase map (h) is mirrored in the line *ω* = *ω*_0_, meaning that for a given pair of singularities, not only the frequency ordering is flipped, but also the topological charges. This evidences that poles and zeros are not simply interchanged when going from a lossy polarizability to an equivalent gainy polarizability. The explanation is that the entire polarizability flips sign, i.e., both the real and imaginary parts, as opposed to complex conjugating which is common in some branches of literature on amplifying nanophotonics, *PT*-symmetry and CPA-lasing [17], [18], [30], [31], [54], [64]. In the framework of the Salisbury screen, the approach *γ* → −*γ* (leading to an amplifying but unphysical polarizability) would interchange perfect absorption for perfect amplification points, *without* rearranging the location of the singularities in phase space. The fact that a correct gain dispersion also changes the real part of the response function is well-known in other fields, such as the field of anomalous dispersion in gain media [51], [52], where the effect is responsible for superluminal light propagation.

### 3.2 Gainy spacers in lossy etalons

**Figure 4:**
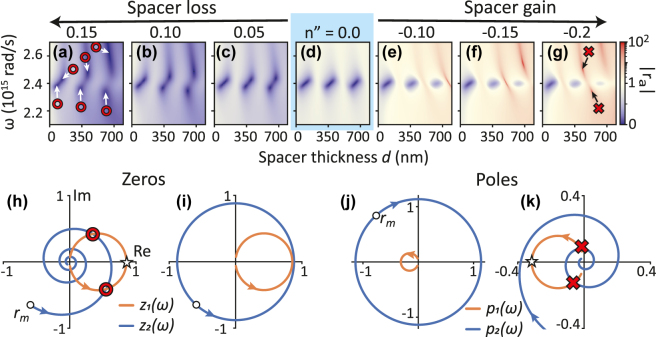
Response of metasurface etalons with non-dispersive loss or gain in the dielectric spacer. The metasurface comprises standard lossy plasmon antennas. (a–g) Evolution with increasing loss resp. gain of the reflection amplitude. The reference case *n*″ = 0 corresponds to panel (d). Absorption singularities appear for *n*″ ≥ 0.05, and are highlighted in (a) with red ◦. Reflection poles only appear for *n*″ ≤ −0.15, indicated in (g) with red × for *n*″ = −0.2. (h–i) resp. (j–k) Complex-plane construction of conditions for reflection zeros resp. poles. Orange (blue) curves correspond to *z*_1_ and *p*_1_ (resp. *z*_2_, *p*_2_). Panel (h) shows how a spacer absorption of *n*″ = 0.15 generates intersections, i.e. perfect absorption points, which do not exist in the case of zero spacer loss (panel i), owing to insufficient antenna density at the chosen *a* = 350 nm. Similarly, panel (k) shows the generation of amplification singularity conditions, which only exist for sufficient spacer gain (here, *n*″ = −0.2). Evaluated for pitch *a* = 350 nm, *V* = 3 × 10^−23^ m^3^ and Au mirror thickness 50 nm. Open black circles in (h,i) resp. (j,k) indicate *r*_*m*_ resp. 1/*r*_*m*_ (starting point of *z*_2_ resp. *p*_2_ at *d* = 0), while the asterisks indicate *z*_1_ resp. *p*_1_ at scatterer resonance *ω* = *ω*_0_. Zeros and pole conditions are indicated by the red circles and crosses.

Next, we explore systems that simultaneously have both gain and loss. A first system one could envision is a standard Salisbury screen (lossy plasmonic particles), but imbuing the spacer medium with gain. We study the dependence of the Salisbury screen response on the imaginary part of the refractive index of the glass spacer layer in Figure 4, ignoring dispersion in the gain. As a starting point we take the case of Figure 3b: A Salisbury screen made with a plasmon lattice that is not quite dense enough to create points of perfect absorption if the spacer has neither gain nor loss (reproduced as panel Figure 4d). To the left of panel (d), in panels (a-c) we consider increasing loss, and to the right, in panels (e-g), increasing gain. Spacer loss/gain is modeled through a non-dispersive imaginary refractive index *n*″, while a constant *n*′ = 1.45 defines the real part. For increasing spacer loss, we already notice for *n*″ = 0.05 that absorption singularity-pairs emerge. Their frequency separation increases with the amount of spacer loss (*n*″) and also with spacer thickness *d*. The latter effect is to be contrasted with Salisbury screens with lossless spacer: If there are singularities, they occur at all successive etalon orders and at identical frequencies, see Figure 3c and d). Turning to the case of gainy spacers in Figure 4e–g, amplification singularities appear for *n*″ = −0.15. Amplification is strongest at the etalon resonance conditions, and pockets of absorption persist. It is obvious that zeros and poles cannot simply be interchanged when *n*″ → −*n*″. Indeed, it is immediately obvious that there is no time reversal symmetry when inverting the sign of *n*″ in the spacer, while maintaining constant particle losses.

For standard plasmonic Salisbury screens, the topological origin of perfect absorption points, and the necessity for them to occur in pairs [28], can be explained by the simple Fabry–Perot interference model Eq. (10). This analysis generalizes to the zeros and poles of amplifying metasurface etalons. Zeros arise from the numerator, and occur when the complex-valued quantities

(11) $$z_{1}\left(\omega \right)=\frac{-r_{a}\left(\omega \right)}{1+2r_{a}\left(\omega \right)}\;\text{and}\;z_{2}\left(\omega ,d\right)=r_{m}e^{2\mathrm{ik}\left(\omega \right)\mathrm{nd}}$$

are equal. Complex functions *z*_1_(*ω*) and *z*_2_(*ω*, *d*) are plotted in Figure 4i for a lossless spacer. The quantity *z*_1_ is solely dependent on the metasurface. When sweeping frequency *ω*, *z*_1_ traces a circle in the complex plane, starting at the origin for zero frequency, returning to it at infinite frequency, while reaching its point farthest from the origin when *ω* = *ω*_0_, where *z*_1_ intersects the real axis. The circle grows in radius with increasing oscillator strength *V*, reaching infinite radius when *r*_*a*_(*ω*_0_) = −1/2. At even stronger scattering, the circle appears on the other side of the imaginary axis. The quantity *z*_2_ instead does not depend on the metasurface, but only on the combination of spacer and mirror. For a lossless spacer it simply traces out a circle in the complex plane of radius *r*_*m*_ centered on the origin, both as function of frequency *ω* and spacing *d*. For the lossless example at hand, there is no crossing between *z*_1_ (orange curve), and *z*_2_ (blue curve) and thus no zero reflection points are expected. For somewhat larger oscillator strength (increased radius *r*_*a*_), intersections will occur in pairs and are revisited when increasing the etalon thickness *d* for each revolution over the blue circle.

We now discuss the generalization to lossy spacers. While the metasurface term *z*_1_ is left unchanged, the term *z*_2_ now changes from a circle of radius *r*_*m*_ to a spiral that spirals inward as the radius decreases with frequency *ω* and etalon spacing *d*. One turn of the spiral is traversed when *n*′*k*_0_ *d* corresponds to a 2*π* phase increment, while the change in radius per turn of the spiral is governed by *n*″. In panel 4h, the spiral starts at the point *r*_*m*_ corresponding to just the mirror, and displays already the first round trip a first pair of intersections, while the second turn generates a second pair, etcetera. The intersections correspond to points of zero reflection, and the frequency at which they occur can be read off from *z*_1_, since each point on the orange circle corresponds to a unique frequency. The matched etalon spacing *d* can then be read off from *z*_2_, since each point on *z*_2_ corresponds to a unique value of the product *ωd*. The first two intersections with *z*_2_ occur at frequencies comparatively close to the plasmon resonance. The intersections at the next higher etalon orders occur increasingly far from resonance. All these observations are in line with the transfer matrix calculations of panel (a). In the case of amplification, the spiral grows outwards, meaning that perfect absorption conditions do not occur.

A similar analysis can be made for poles in the reflectivity, for which we analyze the denominator of the Fabry–Perot interference formula. Setting the denominator to zero is equivalent to satisfying the condition *p*_1_(*ω*) = *p*_2_(*ω*, *d*) for two complex valued quantities

(12) $$p_{1}\left(\omega \right)=r_{a}\left(\omega \right)\;\text{and}\;p_{2}\left(\omega ,d\right)=1/\left(r_{m}e^{2\mathrm{ik}\left(\omega \right)\mathrm{nd}}\right),$$

where again *p*_1_ only depends on the metasurface response, and *p*_2_ only depends on the mirror and spacer. Now *p*_1_ travels a clockwise circle in the complex plane with increasing frequency (panel 4j), centered on the negative real axis and touching the origin. Again, the furthest point of *p*_1_ from the origin occurs at plasmon resonance. For *n*″ = 0, the term *p*_2_ (a circle of radius 1/*r*_*m*_) has a much larger radius than *p*_1_, and the system is very far from emergence of amplification singularities. As gain is introduced (panel k in Figure 4), the term *p*_2_ turns from a circle into an inward spiral. This causes the occurrence of crossings, *i.e.*, reflection poles. The large mismatch in radius between *p*_1_ and *p*_2_ means that significant gain or path length is required: In this example crossings only occur at the second round trip. Higher order intersections occur but only at points very far detuned from plasmon resonance, out of the frequency range considered in our plots. Again, all observations are in line with the (*ω*, *d*) map in panel (g). To summarize, when the time-symmetry is explicitly broken, *i.e.*, introducing loss/gain in the spacer while keeping plasmon losses unchanged, very different conditions for generating zeros and poles are predicted.

### 3.3 Both loss and gain in metasurfaces

**Figure 5:**
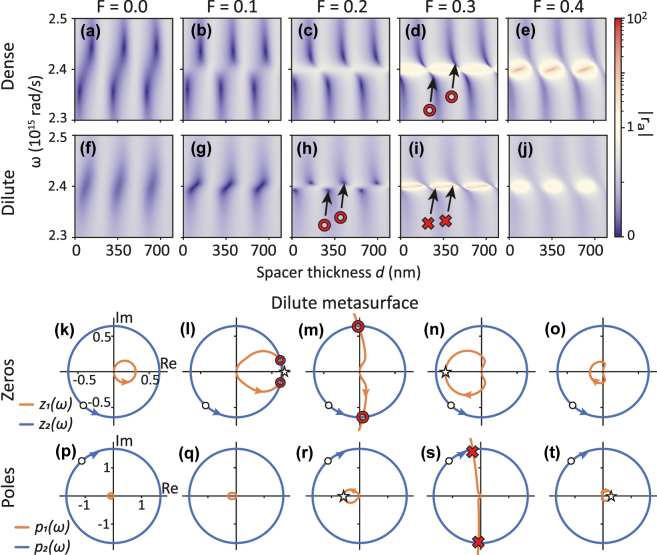
Response of metasurface etalons with gain-plasmon antennas, following the model of Manjavacas [50]. The spacer is lossless. (a–e) Reflectivity amplitude for a dense metasurface (*a* = 250 nm) as function of increasing gain parameter *F*. For *F* = 0 the absorption singularity pairs are far from the plasmon resonance. For increasing *F* the absorption singularities ◦ approach the plasmon resonance *ω*_0_, and ultimately disappear. (f–j) reflectivity amplitude of a dilute metasurface etalon (*a* = 400 nm), without absorption singularities in absence of gain. Absorption singularities are induced by gain (◦ in panel (h), and ultimately are replaced by amplification singularities at larger gain ( × in panel i). (k–o) geometrical construction in the complex plane for reflectivity zeros and poles. Orange (blue) curves correspond to *z*_1_ and *p*_1_ (resp. *z*_2_, *p*_2_). Intersections signify the occurrence of zeros (panel (m)) and poles (panel (s)). Evaluated for *V* = 2 × 10^−23^ m^3^ and Au mirror thickness 20 nm. Open black circles in (k–o) resp. (p–t) indicate *r*_*m*_ resp. 1/*r*_*m*_ (starting point of *z*_2_ resp. *p*_2_ at *d* = 0), while the asterisks indicate *z*_1_ resp. *p*_1_ at scatterer resonance *ω* = *ω*_0_. Zero and pole conditions are indicated by the red circles and crosses.

We finally proceed to the scenario of metasurface etalons with lossless spacers and a loss-gain metasurface, following the model of Manjavacas, where the permittivity function reads *ϵ*(*ω*) = *ϵ*_Drude_(*ω*) + *χ*_gain_(*ω*) [50]. Importantly, Re(*α*) does not immediately flip sign upon increasing population inversion (Figure 2g and h). We take antenna volumes *V* = 2 × 10^−23^m^−3^ and assume the gain resonance *ω*_*g*_ to overlap with the plasmon resonance *w*_0_, and take a line width *γ*_*g*_ = 0.01*ω*_*g*_. Figure 5 considers dense (*a* = 250 nm, panels a–e) and dilute (*a* = 400 nm, panels f–j) lattices. For the dense lattice at hand, Figure 5a shows that in the absence of gain, absorption singularity pairs exist at frequencies quite far away from plasmon resonance. Increasing the gain parameter *F* brings the pairs closer to the resonance, until they disappear for *F* = 0.4. For the dilute lattice without gain, no absorption singularities exist (panel f), but as *F* increases (panels (g-j)), two absorption singularities emerge (just below *F* = 0.1). They annihilate at *F* = 0.3, when two gain singularities have emerged. Again, we can explain the singularity behavior by searching for intersections of the zero functions *z*_1_ and *z*_2_ in panels (a-e), and pole functions *p*_1_ and *p*_2_ in panels (k-o). While for the loss/gain spacer case (Figure 4), the terms *z*_2_ and *p*_2_ (blue curves) were modified by loss/gain, here the changes occur in the metasurface terms *z*_1_ and *p*_1_ (orange curves).

Focusing on the absorption zeros, if one starts with the dilute metasurface, the circle generated by *z*_1_ is too small to generate an intersection with the term *z*_2_ (blue circle of radius *r*_*m*_). Upon pumping, loss compensation causes the metasurface response *z*_1_ to grow in radius, creating a pair of intersections (compare panels k,l,m). Optical gain thus causes pairs of reflection zeros that will occur at all etalon orders. For further increasing gain, the singularities disappear (panel n) owing to the fact that *z*_1_ shrinks again. It should be noted that the sharp, non-trivial dispersion causes the *z*_1_ locus to deviate from the circular shapes that occur for simple Lorentzian antennas. Turning to the occurrence of poles one notices a similar evolution, with the locus of *p*_1_ growing in radius in *k*, reaching a condition where two singularities originate in pairs (panel s), while the gain singularities disappear for even stronger pumping. For both the zero and pole construction, the trajectories of *z*_1_ resp. *p*_1_ change the orientation at which they cross through the real axis at critical *F* values. For the reflection zeros, this is associated with Re[*r*_*a*_] crossing the value 1/2 (flipping the sign of *z*_1_), while for the poles, this reversal occurs when Re[*r*_*a*_]) changes sign, which happens when the metasurface by itself (in absence of the back reflector) goes through its gain singularity (Figure 2g). In summary, in this type of metasurface etalon the addition of gain can induce both perfect absorption points and amplification singularities. Singularities require a critical gain: They disappear both when gain is too low and when gain is too large.

**Figure 6:**
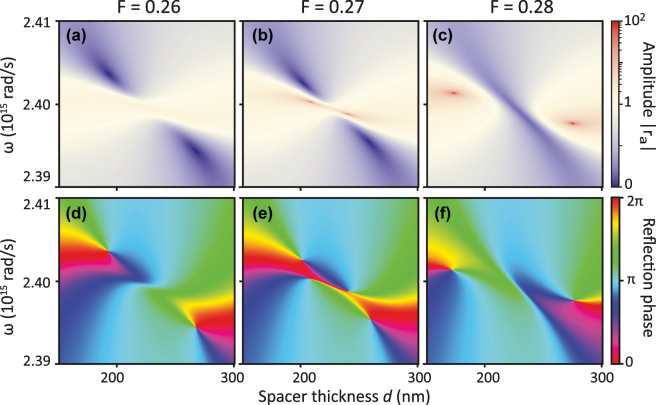
Birth and annihilation of absorption and amplification singularities. We consider the dilute (*a* = 400 nm) amplifying metasurface etalon of Figure 5. (a–c) and (d–f) Reflection amplitude and phase in a small part of *ω* − *d* parameter space for small increments of gain *F*. At *F* = 0.27 (panel b,e), both absorption and gain singularities are present, while in panels (a,d) and (c,f) only zeros resp. only poles occur. Evaluated for *V* = 2 × 10^−23^ m^3^ and Au mirror thickness 20 nm.

For the system with loss and gain, one may wonder if zeros and poles can coexist - or even coalesce - in (*ω*, *d*) space. In Figure 6, we focus on a small region in (*ω*, *d*) space, and very small increments of *F* around the emergence of gain singularities. For *F* = 0.26, the reflectivity amplitude plot (panel a) displays two perfect absorption points, as substantiated by the two oppositely charged phase singularities in the reflection phase (panel d). Here, *z*_1_ and *z*_2_ intersect, but not *p*_1_ and *p*_2_. When increasing gain only very slightly to *F* = 0.27, also *p*_1_ intersects *p*_2_ near resonance, and two amplification singularities co-exist together with the perfect absorption points (panels b, e). Another increment in gain pushes the gain singularities away from each other, while on a trajectory in between the gain singularities, the two absorption singularities approach and annihilate (*F* = 0.28, panels c,f). The co-existence of two types of singularities in parameter space is reminiscent of Ref. [20]. One can ask if this behavior is generic, or if one can construct conditions in which the creation of the amplification singularity pair exactly coincides with the annihilation of the absorption singularity pair. Mathematically, the poles and zeros can only exactly coincide in the case of a perfect etalon, with a perfect mirror |*r*_*m*_| = 1, and when at the same time *r*_*a*_ = −1. The latter solution from Eq. (7) implies a static polarizability *α*_0_(*ω*) = *∞*, which requires not only the usual particle plasmon resonance condition (Re[*ϵ*(*ω*) + 2*ϵ*_host_] = 0) to be fulfilled, but also perfect compensation of the plasmon loss by the gain [65], [66]. While this perfect mirror condition can never be strictly achieved, for very high reflectivity mirrors and antenna’s operated near the polarizability singularity condition, the zero and pole may approach each other very closely in parameter space.

## 4 Discussion

We analyzed the physics of absorption and amplification singularities in reflectivity (in (*ω*, *d*) space) of plasmonic metasurface etalons with amplifying constituents. Several observations stand out. First, replacing plasmon antennas with pure gain counterparts is not simply a time-reversal operation in which loss singularities become gain singularities. Two aspects are at play. First, for pure gain antennas not only the imaginary part of polarizability dispersion flips sign, but also the real part. Hence singularities appear at the same equivalent oscillator strength (|*V*|), but not at the same *ω* − *d* combinations. Second, time-reversing a scattering experiment is generally not equivalent to swapping loss and gain rates, as radiation loss is not inverted in sign. A further set of peculiar observations is that (A) gain can induce perfect absorption, and (B) if gain induces poles in reflection, these conditions of singular reflection only occur at isolated (pairs of) *ω* − *d* points, (C) these generally do not persist indefinitely as gain is increased. The observation that one requires critical gain, and that *more* gain removes singular response, is reminiscent of the physics of critical coupling: Loss, gain, and coupling rates need to be all carefully matched to obtain singular response.

One has to acknowledge that in this conceptual theory work we have used gain parameters that cannot be readily obtained in optics (our values imply gain coefficients up to *g* = 2 × 10^4^ cm^−1^). Nonetheless we argue that the phenomena may be realizable in experiments. Firstly, high gain parameters can be achieved by for instance using perovskite quantum dots [54]. Perovskite Mie scatterers that demonstrate resonant net gain and room temperature lasing have been demonstrated in literature [67], quoting *g* = 3 × 10^4^ cm^−1^ as material gain coefficient. Secondly, our model ignores near field enhancement effects that can increase gain. Lastly, one could envision using waveguiding geometries and local density of states enhancements to make better use of material gain. Indeed, these mechanisms are routinely used in plasmon lattice lasers that show modest lasing thresholds (mJ/cm^2^) at material gain coefficients *g* ∼ 100 cm^−2^. We thus envision that in such systems, just below lasing threshold, application possibilities could open up as amplitude and phase tunable metasurface pixels, where each amplifying metasurface etalon forms a single pixel. The possibility to bring the absorption and amplification singularity pairs extremely close to each other in parameter space means this system can be actively tuned to switch between extreme amplitude enhancement and de-enhancement in a very small window of parameters (*F*, *ω*, *d*).

An open question is what the actual experimental fingerprint will be if you address the reflection poles in experiment. We have evaluated a purely linear model which should break down at the reflection pole conditions: The actual description would need to include nonlinear effects such as gain dynamics, saturation and depletion of the gain, as well as noise [54]. To account for such effects, various works employ rate equation models that incorporate both the plasmonic lattice mode and the gain medium. For the lattice interactions, either FDTD [68], [69], [70], [71] or tight-binding methods [72], [73] can be used, where both routes follow density matrix methods to model the four-level gain medium. Having access to lattice plasmons coupled to gain with spatio-temporal resolution is of interest for the effect of plasmonic near fields on the gain coefficient: The plasmon mode comes with deeply subwavelength spatial and picosecond temporal signatures, and in Refs. [69], [70] it is shown that this leads to highly local gain enhancements. Although the plasmonic gain enhancements are beneficial for the amplification of a probe pulse that harvests the gain, they also lead to spatially/temporally varying gain saturation and depletion of the amplified mode [69], [70]. It remains an interesting but open question how such nonlinearities influence the dynamic behavior of light scattering and lasing near the zero and pole singularities considered in this work. When operated just below lasing threshold, a small signal of probe beam could trigger the onset of lasing [69], which will occur on the pole singularity condition (*ω*, *d*) of the linear model [18]. Otherwise, temporal pulse shaping in the spirit of *virtual gain* might sidestep some of these issues, by accessing the scattering matrix at frequencies away from the real axis [18], [74]. Also, the nonlinear dynamics could open interesting perspectives for these systems as nonlinear, or self-oscillating optical elements [72], [73].
